# Supplementary material for: Culture-Dependent and -Independent Wastewater Surveillance for Multiple Pathogenic Yeasts
Source: J Fungi (Basel). 2025 Jan 23;11(2):86. doi: 10.3390/jof11020086 (PMC11856701; doi:10.3390/jof11020086)
Supplement: Supplementary file 1 [file jof-11-00086-s001.zip › jof-3402796 Supplementary data.pdf]

Supplementary tables

Table S1. Sample locations, coordinates and details.

Sample location (abbreviation)	Coordinates	Details
Bainsvlei treatment plant (BV)	29°06'04.3"S 26°06'54.2"E	Located in western Bloemfontein, collects wastewater from middle-class households, small businesses and surrounding agricultural plots. Universitas hospital complex, including Free State Academic hospital's sewage is treated here.
Woodland Hills treatment plant (WL)	29°07'29.7"S 26°14'46.9"E	Located in northern Bloemfontein close to the N1 national route, collects wastewater from a higher-income community, the military base, including 3 Military hospital, agricultural plots, and large fuel stations on the N1 highway. Thus, includes wastewater from persons passing through Bloemfontein.
Bloemwater treatment plant (BW)	29°07'29.7"S 26°14'46.9"E	Located in central Bloemfontein, collects wastewater from most of the city, including industrial, commercial and residential sources. This plant also has a collection dam for vacuum tankers to dispose of wastewater collected from septic tanks and other sources that do not deposit wastewater directly into established sewer systems. Pelonomi and National District hospitals' wastewater is treated here.
Sterkwater treatment plant (SW)	29°11'22.8"S 26°18'28.8"E	Located in southern Bloemfontein, close to an informal settlement, collects wastewater from agricultural plots, two high-security prisons and low-income to indigent communities.
New Northeast treatment plant (NNE)	29°05'28.3"S 26°19'21.7"E	Located in north-eastern Bloemfontein close to the Bram Fischer International airport, collects wastewater from the airport, agricultural plots and small businesses in the area.
University of the Free State (UFS)	29°06'58.4"S 26°11'26.0"E	A manhole on the university's Bloemfontein campus. Wastewater passing through this point is distributed to the BW treatment plant.

Table S2. Yeast species and their expected band sizes when visualized using gel electrophoresis.

Mutiplex PCR number	Yeast species	Expected band size (bps)
Multiplex 1	*Pichia kudriavzevi*	1159
	Candida dubliniensis	718
	Candida albicans	606
	Candida parapsilosis	490
	Candida auris	331
	Candida glabrata	212
	Candida tropicalis	126
Multiplex 2a	*Clavispora lusitaniae*	377
	Meyerozyma guilliermondii	302
	Kluyveromyces marxianus	203
Multiplex 2b	*Debaromyces hansenii*	818
	Diutina rugosa	689
	Pichia norvegensis	536
	Yarrowia lipolytica	149
Multiplex 3	*Trichosporon asahii*	483
	Trichosporon lactis	480
	Cryptococcus neoformans	392
	Geotrichum candidum	299
	Cryptococcus deneoformans	235
	Cryptococcus gattii	184
	Rhodotorula mucilaginosa	111
