# Supplementary figures and images for: Culture-Dependent and -Independent Wastewater Surveillance for Multiple Pathogenic Yeasts
Source: J Fungi (Basel). 2025 Jan 23;11(2):86. doi: 10.3390/jof11020086 (PMC11856701; doi:10.3390/jof11020086)

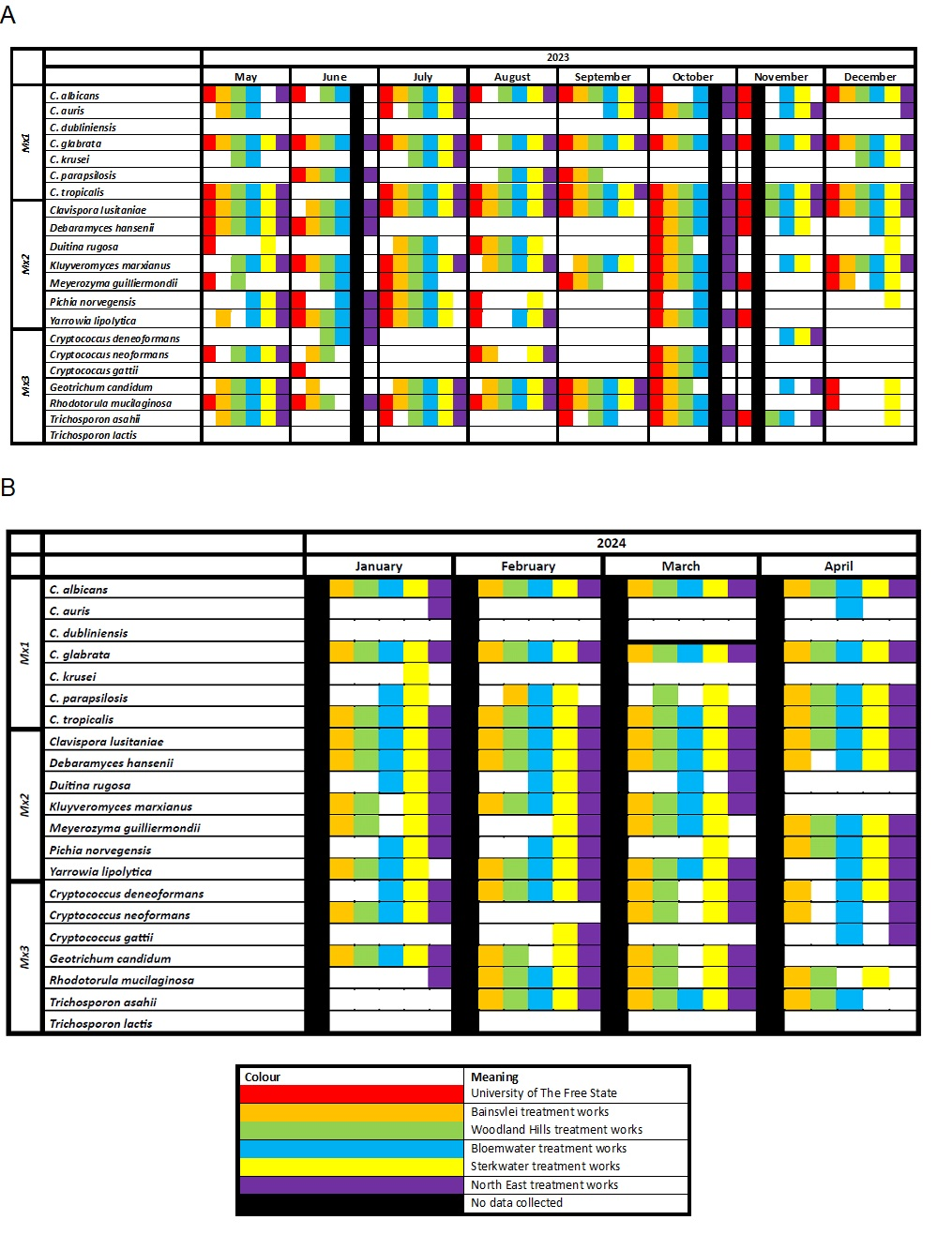

Supplement: Supplementary file 1 [file jof-11-00086-s001.zip › Figure S1.tiff]
